# Practicing what we preach: developing a data sharing policy for the *Journal of the Medical Library Association*

**DOI:** 10.5195/jmla.2018.431

**Published:** 2018-04-01

**Authors:** Kevin B. Read, Liz Amos, Lisa M. Federer, Ayaba Logan, T. Scott Plutchak, Katherine G. Akers

**Affiliations:** Data Services Librarian and Data Discovery Lead, NYU Health Sciences Library, New York University School of Medicine, 577 First Avenue, New York, NY 10016; Librarian, National Information Center on Health Services Research and Health Care Technology, National Library of Medicine, Bethesda, MD; Research Data Informationist, NIH Library, National Institutes of Health, Bethesda, MD; Research and Education Informationist, Libraries, Medical University of South Carolina, Charleston, SC; Director of Digital Data Curation Strategies, Lister Hill Library of the Health Sciences, University of Alabama, Birmingham, AL; Editor-in-Chief, *Journal of the Medical Library Association*

## Abstract

Providing access to the data underlying research results in published literature allows others to reproduce those results or analyze the data in new ways. Health sciences librarians and information professionals have long been advocates of data sharing. It is time for us to practice what we preach and share the data associated with our published research. This editorial describes the activity of a working group charged with developing a research data sharing policy for the *Journal of the Medical Library Association.*

The sharing of research data underlying scholarly literature is essential for research transparency. Health sciences librarians and information professionals strongly believe in the openness of information and have long been advocates of data sharing [1–3]. Members of our community spend considerable time developing and implementing services and policies to encourage researchers to share their data [4]. Among the many reasons for data sharing, access to research data can improve the integrity of the research process by allowing others to reproduce research results or analyze the data in new ways [5]. Some peer-reviewed journals such as *PLOS ONE* [6] have taken the lead in requiring authors to share their data if they want to publish in these journals, whereas other journals like the *New England Journal of Medicine* have yielded to their research communities’ concerns around data sharing and released less stringent data sharing guidelines [7]. The latter has justifiably disappointed many open data advocates who wish to see biomedical research improved by greater transparency and access to research data [8, 9].

To date, the *Journal of the Medical Library Association (JMLA)* has not enacted policies related to sharing research data. However, as discussions around data in health sciences librarianship continue to build and more journals require data sharing as a precondition for publishing, it is time for librarians to practice what we preach and share the data and documentation associated with our published research. As we encourage our user communities to comply with data sharing practices, we must adhere to these practices as well.

In May 2017, the editor-in-chief of the *JMLA* tasked a working group with developing a data sharing policy for the journal. To develop this data sharing policy, the working group (1) contacted authors of articles recently published in the *JMLA* to assess their willingness or ability to share their data and to understand their concerns about data sharing, (2) reviewed data sharing policies of other peer-reviewed journals, and (3) is working to align *JMLA* policy with recommendations of the Research Data Alliance (RDA) Data Policy Standardisation and Implementation Working Group [10]. The group’s goal is to develop a data sharing policy that promotes the rigor and reproducibility of research described by articles published in the *JMLA,* while also imposing a reasonably minimal burden on authors, peer reviewers, and editorial team members.

## FEEDBACK FROM JOURNAL OF THE MEDICAL LIBRARY ASSOCIATION AUTHORS

To inform the development of a *JMLA* data sharing policy, the group contacted twenty-one authors of research articles or case studies published in the *JMLA* in 2016. After explaining our intention to develop a *JMLA* data sharing policy, we asked the authors if they would send our working group the data that would be needed to reproduce the results described in their articles. If authors could not or chose not to share the data with us, we asked them to provide an explanation. Finally, we asked authors if they had concerns about the implementation of a *JMLA* data sharing policy. In several cases we had to search, sometimes unsuccessfully, for authors’ new email addresses if we received notice that they had left their former institutions. Nonresponses to our initial email request were followed up by a reminder email.

We heard back from 15 authors, 11 of whom sent us partial or complete datasets, sometimes accompanied by supporting documentation or computer code. Responding authors who did not send us their data gave the following reasons: the protocol approved by their institutional review board (IRB) specifically forbade data sharing (n=1), the statistician who managed the data had left the institution (n=1), data could not be located (n = 1), and article coauthors were not comfortable with sharing the data (n=1). We note that had there been a journal data sharing policy in place at the time these manuscripts were planned, prepared, or submitted, at least some of these barriers to data sharing might not have existed. For instance, authors’ plans to share data could have been approved by the IRB before the study took place, and data could have been submitted to a trustworthy repository and might not have been lost.

**Figure 1:**
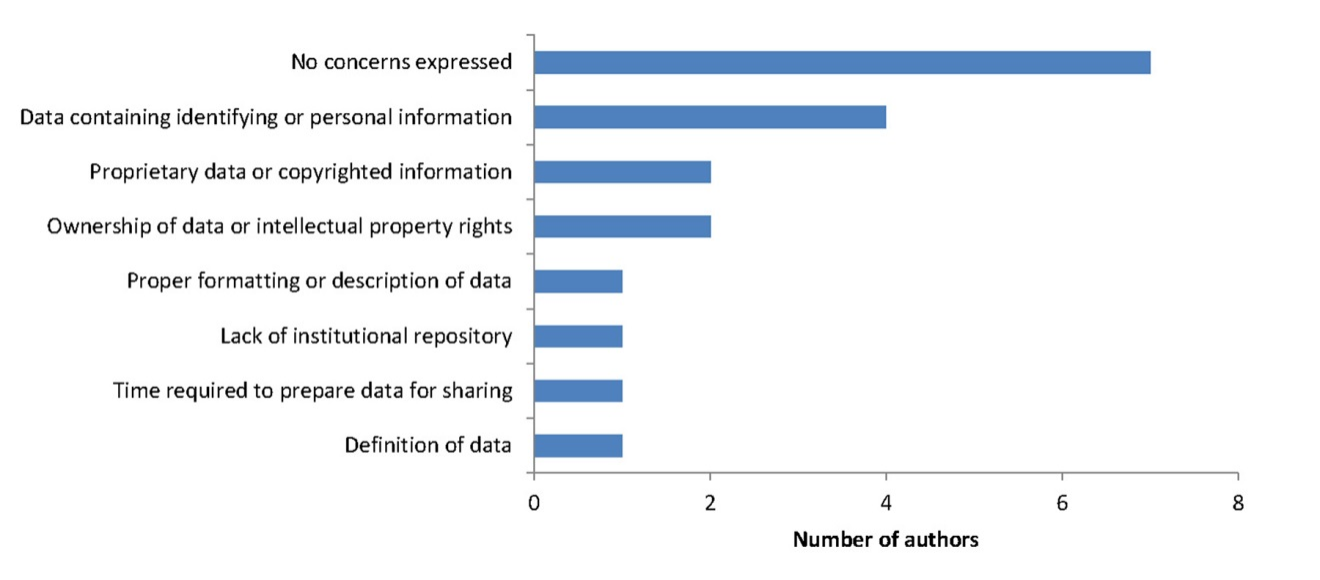
Concerns of authors regarding a potential *Journal of the Medical Library Association* data sharing policy Of the 15 authors who responded to our email, 7 expressed no concerns, whereas 8 expressed 1 or more concern about data sharing.

Of the authors who responded to our email, approximately half did not express any concerns regarding a potential *JMLA* data sharing policy (Figure 1), and many (n=8) explicitly voiced support for such a policy. Among those who expressed concerns about a journal data sharing policy, the most common concern was how the policy would address sharing data that contained identifying or personal information. Some of these authors stated that sharing data would require modifications to their IRB protocols, whereas others worried about the lack of standards or procedures in the library and information science field for determining what and how identifying information should be removed. Other authors were concerned about data ownership and intellectual property rights: Would authors still retain rights to the data? How would access be controlled? Would the policy address what other people would be allowed to do with an author’s data? Some authors pointed out that data sharing would be difficult or impossible in cases in which some data were proprietary or included copyrighted materials. Finally, some authors asked how data would need to be formatted or described to be usable or understandable by others, wondered where authors without institutional repositories were to deposit their data, noted the not negligible time needed to prepare a dataset and its documentation for sharing, and wanted clarification on what we meant by “data.”

Indeed, “data” can conceivably encompass a great number of different types of research products, including spreadsheets, text files, interview recordings and transcripts, survey and assessment instruments, images, videos, and computer codes and scripts, as well as documents describing the data or how it was collected (e.g., study protocols, data dictionaries, codebooks, readme files). These concerns have been duly noted by our working group and are being taken into serious consideration as we develop the policy. Each issue raised by the authors provides valuable information that is helping shape policy details and language.

## REVIEW OF EXISTING JOURNAL DATA SHARING POLICIES

To familiarize ourselves with the language used in other peer-reviewed journals’ data sharing policies, we reviewed policies addressing data accessibility from journals published by PLOS [6], *Gigascience* [11], *Science* [12], journals published by the Nature Publishing Group [13], and Nature Publishing Group’s *Scientific Data* in particular [14]. The goal of this exercise was to assess different journal policies ranging from those that firmly require data sharing in order to publish (i.e., *PLOS ONE*) to those that only ask for a data availability statement (i.e., *Nature*). For each policy, we focused on the journal’s definition of data, acceptable methods for data sharing, permitted reasons for not sharing data, and any required technical specifications (e.g., file formats, documentation). These details have provided valuable insight into how to structure and word the data sharing policy for the *JMLA.*

## ALIGNMENT WITH RESEARCH DATA ALLIANCE DATA POLICY STANDARDISATION AND IMPLEMENTATION RECOMMENDATIONS

To ensure that the *JMLA* data sharing policy aligns with larger efforts in scholarly publishing, we have kept abreast of the conversations of the RDA Data Policy Standardisation and Implementation Working Group, which includes representatives from several major publishers and publishing organizations and aims to establish common frameworks for journal data policies that can be used by journals across disciplines [10]. As we align our policy with RDA guidelines, we will be asking *JMLA* authors to adhere to policies consistent with those of other journals to which they may be submitting.

## FUTURE STEPS

After gathering feedback from *JMLA* authors, reviewing existing journal data sharing policies, and being informed by RDA Data Policy Standardisation and Implementation Working Group discussions, we created a list of several questions that our working group must answer to develop a *JMLA* data sharing policy that not only compels authors to share the data underlying their research results, but also addresses common data sharing concerns held by researchers and practitioners in our field. These questions pertain to what our definition of data is, what types of articles to which the policy will apply, where data should be deposited, when data should be made available, what acceptable reasons are for restricting access to data, and what Frequently Asked Questions or other guidance we should provide to help authors comply with the policy. We also plan to converse with and solicit feedback from members of the *JMLA* Editorial Board, Medical Library Association (MLA) Data Special Interest Group, MLA Research Section, and MLA Board of Directors. Our goal is to officially implement a *JMLA* data sharing policy in 2018.

## FINAL THOUGHTS

We hope that health sciences librarians will be excited by the prospect of sharing the data associated with their published articles in accordance with the new *JMLA* data sharing policy and leading by example for their user communities. As the corpus of health sciences library and information research data grows under this policy, so will the integrity of our research processes and findings. The more we share data and encourage others to do the same, the better chance we have of increasing collaboration within and outside our domain, making new discoveries, and becoming a leading field in research data sharing practices. Furthermore, by experiencing the process of preparing our own data to be shared, we can learn more about the challenges that our users face when asked to share their data. This newfound knowledge will make us better educators, advocates, and guides for improving the sharing of biomedical data by researchers at our own institutions. To communicate your ideas for or concerns with a *JMLA* data sharing policy, please contact *JMLA* Editor-in-Chief Katherine Akers, at jmla@journals.pitt.edu.
